# Nighttime kidney transplantation is associated with less pure technical graft failure

**DOI:** 10.1007/s00345-015-1679-0

**Published:** 2015-09-14

**Authors:** Denise M. D. Özdemir-van Brunschot, Andries J. Hoitsma, Michel F. P. van der Jagt, Frank C. d’Ancona, Rogier A. R. T. Donders, Cees J. H. M. van Laarhoven, Luuk B. Hilbrands, Michiel C. Warlé

**Affiliations:** Division of Vascular and Transplant Surgery, Department of Surgery, Radboud University Medical Center Nijmegen, Geert Grooteplein-Zuid 10, 6525 GA Nijmegen, The Netherlands; Department of Nephrology, Radboud University Medical Center, Geert Grooteplein-Zuid 10, 6525 GA, Nijmegen, The Netherlands; Department of Urology, Radboud University Medical Center, Geert Grooteplein-Zuid 10, 6525 GA Nijmegen, The Netherlands; Department of Health Evidence, Radboud University Medical Center, Geert Grooteplein-Zuid 10, 6525 GA Nijmegen, The Netherlands

**Keywords:** Day, Graft survival, Night, Renal transplantation, Technical failure

## Abstract

**Purpose:**

To minimize cold ischemia time, transplantations with kidneys from deceased donors are frequently performed during the night. However, sleep deprivation of those who perform the transplantation may have adverse effects on cognitive and psychomotor performance and may cause reduced cognitive flexibility. We hypothesize that renal transplantations performed during the night are associated with an increased incidence of pure technical graft failure.

**Methods:**

A retrospective analysis of data of the Dutch Organ Transplant Registry concerning all transplants from deceased donors between 2000 and 2013 was performed. Nighttime surgery was defined as the start of the procedure between 8 p.m. and 8 a.m. The primary outcome measure was technical graft failure, defined as graft loss within 10 days after surgery without signs of (hyper)acute rejection.

**Results:**

Of 4.519 renal transplantations in adult recipients, 1.480 were performed during the night. The incidence of pure technical graft failure was 1.0 % for procedures started during the night *versus* 2.6 % for daytime surgery (*p* = .001). In a multivariable model, correcting for relevant donor, recipient and graft factors, daytime surgery was an independent predictor of pure technical graft failure (*p* < .001).

**Conclusions:**

Limitation of this study is mainly to its retrospective design, and the influence of some relevant variables, such as the experience level of the surgeon, could not be assessed. We conclude that nighttime surgery is associated with less pure technical graft failures. Further research is required to explore factors that may positively influence the performance of the surgical team during the night.

## Introduction

As the diagnosis of brain death and decisions around potential multiorgan donors are usually made during office hours, multiorgan donor surgery is usually started during the evening or night [1]. As allocation, organ transport and preparation of the recipient (including cross-match), in general, take 12–19 h [2, 3], most kidney transplantations are performed during office hours. However, when all preparations for surgery are completed in the late evening or very early morning hours, delay of surgery until office hours would result in an unnecessary prolongation of cold ischemia time (CIT). CIT has a major influence on postoperative graft function; it is associated with an increased risk of delayed graft function (DGF), primary nonfunction (PNF), acute rejection and even increased mortality [4–6]. DGF can result in the delayed diagnosis of rejection, increased risk of graft failure and prolonged hospitalization [5]. To reduce the deleterious influences of prolonged CIT, a significant number of kidney transplantations are performed during the night.

However, younger surgeons are becoming more critical regarding nighttime work and their own quality of life. Also evidence exists showing that sleep deprivation may have adverse effects on cognitive and psychomotor performance [7–9]. Moreover, it may impair working memory and cognitive flexibility, and sleep-deprived subjects tend to make risky decisions [10, 11]. This raises the question whether nighttime surgery is associated with an increased risk of pure technical graft failures. Four studies previously assessed the impact of nighttime surgery on surgical complications and kidney allograft survival [12–15]. One study, performed by Fechner et al, observed a higher incidence of graft failure during the night [14]. This finding could not be confirmed in the three other studies. However, all studies were single center and contained relatively small patient series, which may have introduced a type II error.

Therefore, we performed a cohort study with data obtained from the Dutch Organ Transplant Registry to evaluate whether kidney transplantation performed during the night is associated with technical graft failure.

## Patients and methods

### Patients

We used the data of a consecutive series of transplantations with deceased donor kidneys in adult recipients performed from January 1, 2000, to December 31, 2013. Data were obtained from a prospectively maintained electronic database by the Dutch Organ Transplant Registry (NOTR, Nederlandse Transplantatie Stichting, Leiden, The Netherlands).

We identified the following factors as possible confounders: donor gender, age and body mass index (BMI), donation after brain death (DBD) or circulatory death (DCD), hypertension of the donor, kidney side, number of renal arteries, start cold perfusion time, recipient gender, age and BMI, smoker/nonsmoker, vascular status of the recipient, number of dialysis days, previous renal transplants, first warm ischemia time (WIT1), WIT2 and CIT and center of transplantation.

WIT1 was defined as the time from cessation of the blood circulation to the renal allograft to start of cold perfusion. CIT was defined as time between start of cold perfusion and removal of the renal allograft from ice. Second warm ischemia time (WIT2) was defined the time between placement of the renal allograft into the iliac fossa of the recipient, until revascularization of the kidney.

### Outcome measures

In line with existing literature, daytime surgery was defined as start of surgery between 8 a.m. and 8 p.m. and nighttime surgery was started between 8 p.m. and 8 a.m. [13, 14]. Surgery was started after the routine preoperative workup was completed, and the operation room and operating team were available. PNF was defined graft loss despite adequate initial perfusion (*e.g.,* ultrasound proven), while nonviable kidneys (NVK) were defined as never-functioning renal allografts with poor perfusion from the beginning. In many cases of graft failure due to PNF and NVK, it was difficult to differentiate between pure technical graft failures and graft failures due to poor organ quality. Therefore, the primary outcome measure was pure technical graft failure, defined as graft loss within 10 days after the transplantation without signs of (hyper)acute rejection, excluding cases of PNF and NVK. Nonimmunological graft failure was defined as graft loss within 10 days without signs of (hyper)acute rejection, including cases of PNF and NVK, this was a secondary outcome measure. In cases with vascular or urological problems in which the renal allograft could be saved by reintervention, the cases were not considered as technical failures. (hyper)Acute rejection with graft loss within 10 postoperative days was referred to as graft failure due to immunological causes.

### Statistical analysis

Statistical analyses were performed using SPSS (SPSS Inc., version 20, Armonk, NY). Demographic data were reported as means and standard deviation (SD) for continuous variables; categorical variables were reported as number and percentages. Student’s *t* test was used to compare normally distributed variables, and *χ*^2^ for categorical variables. For all possible confounding factors, logistic regression was performed to identify factors associated with time of start of the procedure and with pure technical graft failure. All factors associated with time of start of the procedure and pure technical graft failure (defined as *p* ≤ 0.05) were included in the multiple regression analysis. Graft survival was estimated using the Kaplan–Meier method, and groups were compared with log-rank tests. For all analyses, statistical significance was set at *α* = 0.05.

## Results

### Baseline characteristics

**Fig. 1:**
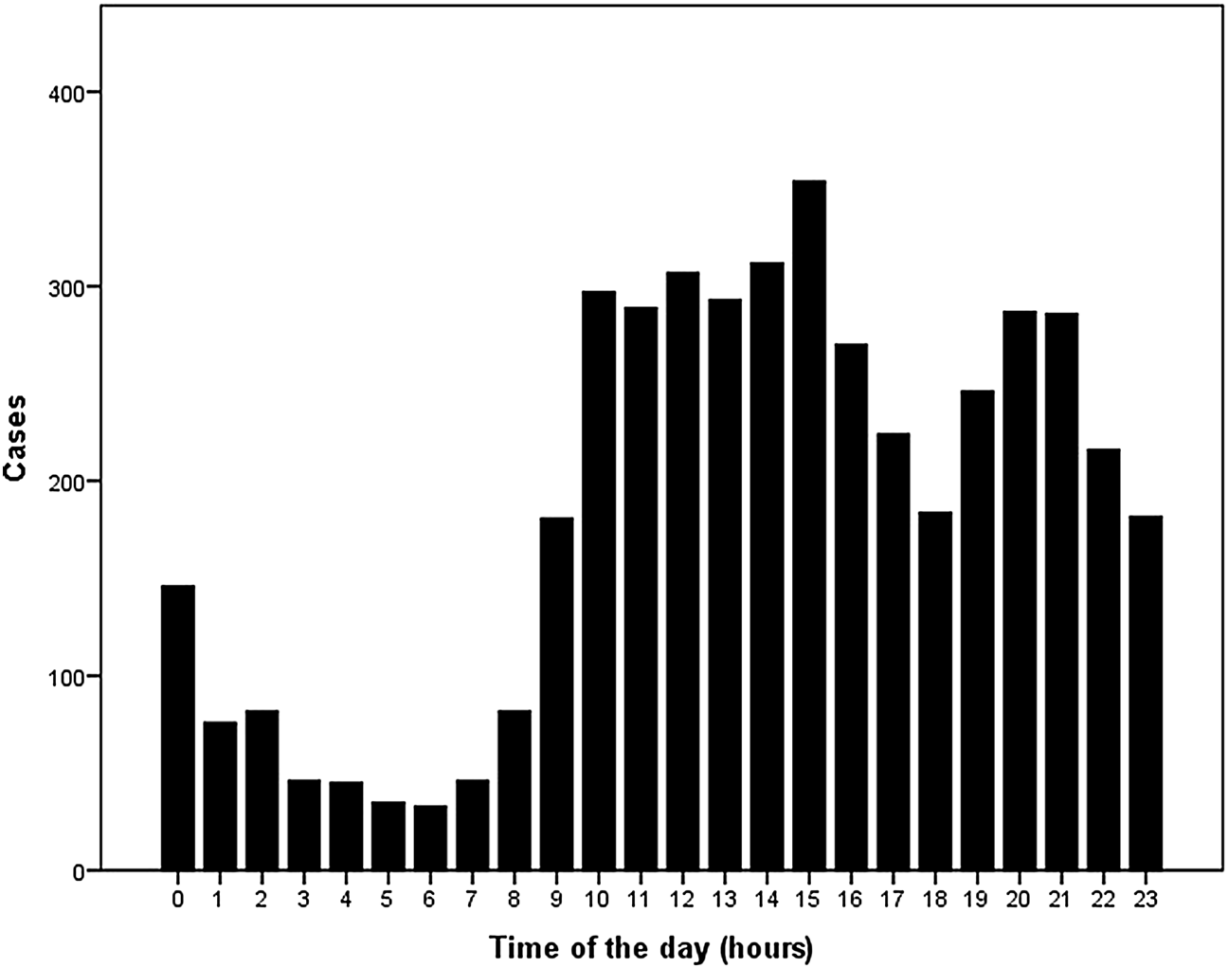
Distribution of timing of transplantations

In total, 4.519 consecutive renal transplantations from deceased donors in adult recipients were included in the analysis. Baseline characteristics for donor and recipients, and surgical parameters are shown in Table 1. A total of 3.039 procedures were performed during daytime, between 8 a.m. and 8 p.m. The distribution of the start of surgery time is shown in Fig. 1. During the night, the contribution to male donors, DBD kidneys, right kidneys and retransplantations was significantly higher. CIT was significantly longer for transplantations during the nightly hours.

**Table 1:** Recipient and donor characteristics and surgical parameters

	All (*n* = 4.519)	Per time group		
		8 p.m.–8 a.m. (*n* = 1.480)	8 a.m.–8 p.m. (*n* = 3.039)	*p* value
*Donor characteristics*				
Age (year)	48.1 (15.4)	47.7 (15.2)	48.2 (15.5)	.30
Male gender	2323 (51.4 %)	796 (53.8 %)	1527 (50.2 %)	.03
BMI (kg/m^2^)	25.0 (4.4)	25.2 (4.5)	24.9 (4.4)	.06
DBD	2695 (59.6 %)	935 (63.2 %)	1760 (57.9 %)	.00
Right kidney side	2220 (49.1 %)	768 (51.9 %)	1452 (47.8 %)	.01
Multiple arteries	926 (20.6 %)	299 (20.3 %)	627 (20.8 %)	.74
*Recipient characteristics*				
Age (year)	51.1 (15.0)	51.0 (15.1)	51.1 (15.0)	.85
Male gender	2689 (59.5 %)	866 (58.5 %)	1823 (60.0 %)	.34
BMI (kg/m^2^)	25.2 (4.5)	25.2 (4.5)	25.3 (4.5)	.44
Smoker	689 (24.0 %)	226 (19.4 %)	463 (19.4 %)	.98
Diabetes mellitus	637 (17.6 %)	222 (18.8 %)	415 (17.0 %)	.20
History of vascular event	487 (15.2 %)	159 (13.2 %)	328 (13.2 %)	.99
Duration dialysis (days)	1546.3 (982.8)	1572.9 (1023.6)	1533.3 (962.2)	.21
*Surgical parameters*				
Retransplantation	740 (16.4 %)	273 (18.4 %)	467 (15.4 %)	.01
WIT1 (NHB) (min)	19.4 (7.7)	20.0 (7.9)	19.1 (7.5)	.02
WIT2 (min)	34.6 (13.6)	34.8 (14.8)	34.5 (12.9)	.57
CIT (min)	1058.0 (375.5)	1105.9 (400.7)	1034.6 (360.4)	.00
*Graft outcome*				
Technical failure^a^	93 (2.1 %)	15 (1.0 %)	78 (2.6 %)	.00
Vascular		12 (.8 %)	49 (1.6 %)	.03
Urological		3 (.2 %)	5 (.2 %)	.77
Other		0 (.0 %)	24 (.8 %)	.00
Technical failure^b^	183 (4.0 %)	49 (3.3 %)	134 (4.4 %)	.08
PNF		27 (1.8 %)	40 (1.3 %)	.18
NVK		7 (.5 %)	16 (.5 %)	.17

*BMI* body mass index, *CIT* cold ischemia time, *DBD* donation after brain death, *DCD* donation after cardiac death, *WIT1* first warm ischemia time, *WIT2* second warm ischemia time

^a^Defined as technical failure excluding PNF and NVK

^b^Defined as technical failure including PNF and NVK

### Graft function and technical graft failure: impact of timing of surgery

The overall incidence of pure technical graft failure was 2.1 % (93 cases; Table 1). The incidence of pure technical graft failure was 1.0 % when surgery was performed during nighttime *versus* 2.6 % during daytime (*p* < .01).

A significant association between daytime surgery and pure technical graft failure was observed (*p* < .00), Table 2. In most cases, the primary cause of pure technical graft failure was vascular; however, in .2 %, urinary obstruction or ureteral necrosis ultimately led to the removal of the graft.

**Table 2:** Univariate and multivariate analyses for pure technical graft failure

Parameters	Univariate analysis		Multivariate analysis	
	OR (95 % CI)	*p* value	OR (95 % CI)	*p* value
Time of surgery	2.573 (1.475–4.487)	.001	3.220 (1.7232–5.984)	.00
*Donor characteristics*				
Age (year)	.990 (.977–1.002)	.110	.991 (.977–1.005)	.22
Male gender	.831 (.549–1.257)	.380	–	–
BMI (kg/m^2^)	1.002 (.957–1.049)	.923	–	–
DBD	1.817 (1.202–2.746)	.005	1.157 (.539–2.483)	.71
Right kidney side	1.512 (.996–2.295)	.053	1.433 (.924–2.221)	.11
Multiple arteries	1.291 (.801–2.081)	.295	–	–
Start cold perfusion^a^	1.752 (1.160–2.647)	.008	1.783 (1.143–2.782	.01
*Recipient characteristics*				
Age (year)	.989 (.976–1.002)	.092	.988 (.974–1.003)	.12
Male gender	.884 (.579–1.351)	.570	–	–
BMI (kg/m^2^)	1.024 (.978–1.073)	.307	–	–
Smoker	.830 (.444–1.550)	.558	–	–
Diabetes mellitus	.828 (.433–1.581)	.567	–	–
History of vascular event	1.299 (.694–2.430)	.413	–	–
Duration dialysis (days)	1.000 (1.000–1.000)	.645	–	–
*Surgical parameters*				
Retransplantation	1.802 (1.125–2.887)	.014	1.840 (1.117–3.031)	.02
WIT1 (DBD) (min)	1.027 (1.009–1.044)	.003	1.021 (.991–1.053)	.00
WIT2 (min)	1.020 (1.009–1.032)	.000	1.021 (1.009–1.034	.00
CIT (min)	1.000 (1.000–1.001)	.071	1.000 (1.000–1.001)	.44

*BMI* body mass index, *CIT* cold ischemia time, *DBD* donation after brain death, *DCD* donation after cardiac death, *WIT1* first warm ischemia time, *WIT2* second warm ischemia time

^a^Divided in 2 periods: 8 a.m.–8 p.m. and 8 p.m.–8 a.m.

For each center, the incidence of pure technical graft failure was (slightly) higher for daytime surgery with a significant difference for two centers (no. 4 and 7; *p* ≤ 0.05).

The incidence of (hyper)acute rejection with graft loss within 10 postoperative days was, respectively, .2 % for daytime and .3 % for nighttime surgery, which was not a significant difference (p. 51).

### Graft survival

**Fig. 2:**
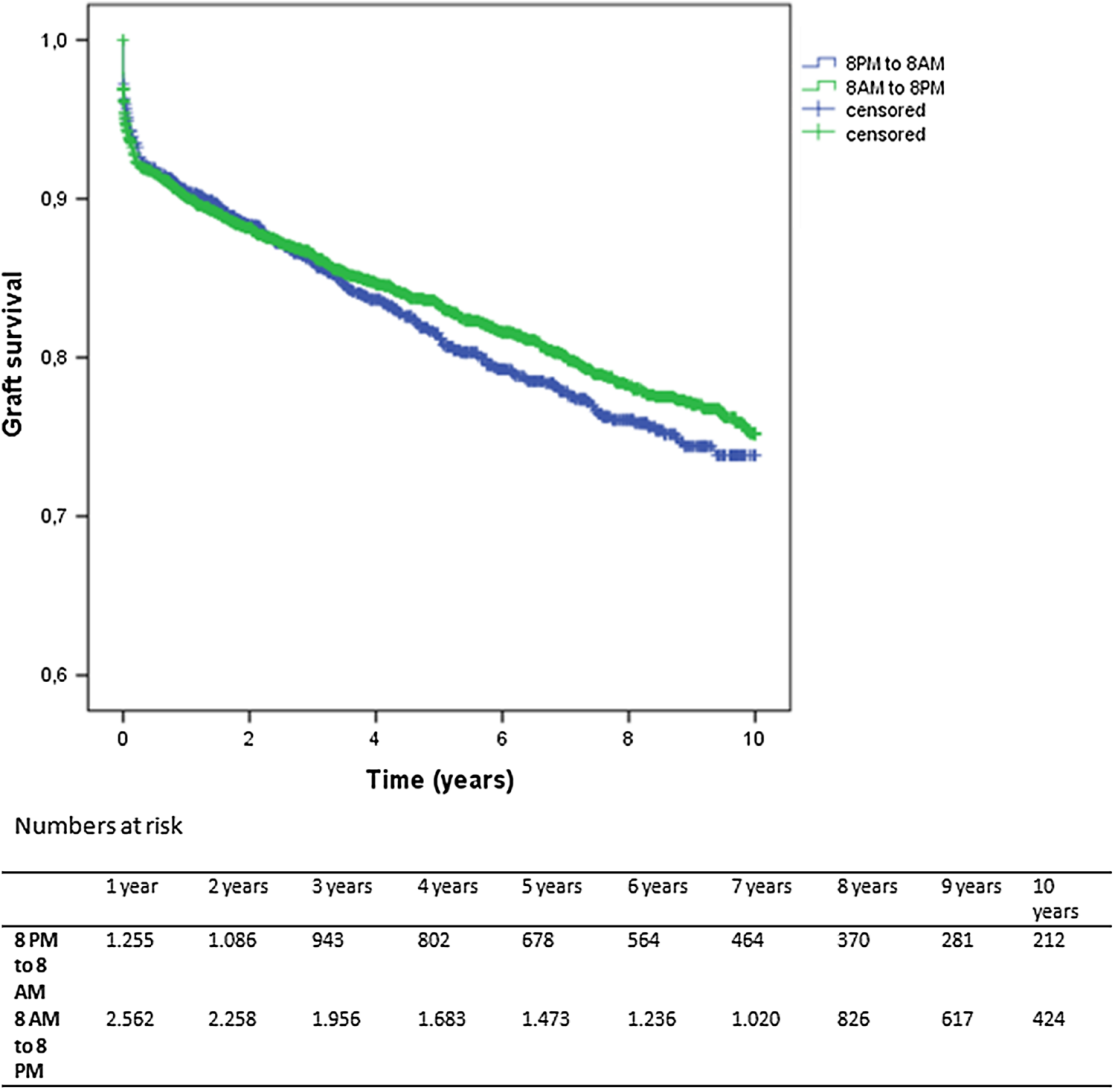
Long-term graft survival for daytime and nighttime surgery (log-rank test .902 *p* = .342)

Long-term graft survival for procedures performed during the day or night are shown in Fig. 2. No significant difference was found between kidneys transplanted during daytime and nighttime (log-rank test .988 *p* = .320).

## Discussion

So far, four studies have been performed addressing the impact of nighttime surgery on complications and allograft function in renal transplantation [12–15]. Fechner et al. observed more vascular complications and higher risk of graft failure when surgery was started during nightly hours, defined as 8 p.m.–8 a.m. [14]. In 3 other studies, nighttime surgery was not associated with decreased graft survival. Additionally, studies in patients undergoing liver transplantation or hip surgery did not demonstrate a significant impact of nighttime surgery on graft outcome or complications [16–19].

Data regarding the influence of overnight surgical activity on complications of next-day surgery and conversion to open surgery are not univocal; some authors could not demonstrate any impact, while Rothschild et al. observed an increased complication rate when next-day surgical procedures were performed by physicians with sleep duration below 6 h [8, 20, 21].

In other professions, the influence of fatigue is more pronounced: less psychomotor vigilance and more lane deviation in a driving simulator for police officers [22], more procedural and tactical decision errors for pilots [23] and more critical incidents (crashes, near-crashes or crash-relevant conflicts) in commercial vehicle drivers [24].

Gastrointestinal or trauma surgery is usually performed during the night to prevent deterioration of the patients’ condition (*e.g.,* due to septic shock or hypovolemia). In contrast, renal transplantations are performed during the night in order to limit CIT. Our observation that nighttime surgery is associated with less pure technical graft failures is remarkable. A possible explanation may be that recipients with a poorer physical condition were postponed to the next morning; however, this is not supported by our data as recipient characteristics (*i.e.,* age, male gender, BMI, smoking status, diabetes mellitus, history of vascular events and time on dialysis) did not differ significantly. Moreover, significantly more transplants with kidneys from DBD donors and retransplants have been performed during the night. Altogether, our data suggest that other factors than those related to the patient and kidney (*i.e.,* factors related to the surgeon and the team) may play an important role. Since organ preservation pumps are only scarcely used in the Netherlands, this could not have had a relevant impact on our data.

In general, the assumption can be made that prior to the nightly renal transplantation, the transplant surgeons had worked a full day. A possible explanation for our findings is the fact that during daytime residents and fellows are being trained. During nightly hours, the senior transplant surgeons might tend to perform the procedure him/herself. Data on the surgeons’ experience were not available in the Dutch Organ Transplant Registry. Furthermore, many surgeons would recognize that during nightly hours the surgical team is less distracted by pagers, disturbance from outside and team shifts, which allows more focus on the surgery. A more “quiescent” operation room during nightly hours has been demonstrated to have a positive impact on surgical performance [25]. It has also been shown that interruptions are correlated with surgical errors [25, 26].

Moreover, there are physiological changes during night hours. Surgeons working during night shifts have decreased heart rate variability and a significant increase in pulse rate, indicating sympathetic dominance in the autonomic nervous system [27]. This may imply more alertness during the procedure.

Although our results show a lower rate of pure technical graft failure for nighttime transplantations, there are also clear disadvantages of working during the nightly hours. Studies have shown that night shift work is associated with increased risk of obesity, metabolic syndrome and some types of malignancies, *e.g.,* colorectal and breast cancer [28–30]. This is probably caused by exposure to light during the night, which disturbs the circadian rhythm [30].

### Strengths and limitations

A major strength of our study is the number of included patients; to our knowledge, this is the largest cohort regarding the influence of surgery during nightly hours on graft outcome in kidney transplantation. Moreover, the database was prospectively maintained by the Dutch Transplantation Foundation.

There are also some limitations. First, data regarding possible confounders, *e.g.,* experience of the surgeon, time since last sleep and length of shift, are not included in the database. Also, we have no data by whom (resident, fellow or staff surgeon) surgery was performed. Secondly, the definition of pure technical graft failure is challenging. However, early graft failure is a devastating complication for the recipient and therefore a highly relevant primary endpoint. Early graft failure is associated with poor patient survival [31, 32] and worse quality of life [33]. To overcome this, a separate analysis was performed for nonimmunological graft failure. As many cases of PNF and also NVK may have been due to poor organ quality or prolonged CIT, the association between nonimmunological graft failure and daytime surgery was more obvious after excluding these cases. In our analysis, CIT was indeed longer for allograft failure due to PNF, when compared to CIT of kidneys which failed due to other technical causes. This did not apply for CIT of graft failure due to NVK.

Another possible factor which could have influenced sleep deprivation is the centers’ policy regarding cold ischemia time. In center 5, which performed 52 % of the kidney transplantations during the night, the shortest mean cold ischemia time was observed (740 min).

Another limitation is the fact that the registration of postoperative complications was insufficient and there could not be analyzed reliably.

## Conclusion and implications for the future

In conclusion, renal transplantations during nightly hours (8 p.m.–8 a.m.) may have a beneficial impact on the pure technical graft failure rate. This supports the current practice in many transplant centers, performing nightly procedures to minimize cold ischemia time. Further research is required to explore factors that may positively influence the performance of the surgeon and his/her team during the night. This knowledge may help to improve the performance of the surgical team during daytime surgery.

## Abbreviations

BMI: Body mass index
CIT: Cold ischemia time
DBD: Donation after brain death
DCD: Donation after circulatory death
DGF: Delayed graft function
WIT1: First warm ischemia time
WIT2: Second warm ischemia time
